# Co-protoporphyrin IX and Sn-protoporphyrin IX inactivate Zika, Chikungunya and other arboviruses by targeting the viral envelope

**DOI:** 10.1038/s41598-018-27855-7

**Published:** 2018-06-28

**Authors:** Romulo L. S. Neris, Camila M. Figueiredo, Luiza M. Higa, Daniel F. Araujo, Carlos A. M. Carvalho, Brunno R. F. Verçoza, Mariana O. L. Silva, Fabiana A. Carneiro, Amilcar Tanuri, Andre M. O. Gomes, Marcelo T. Bozza, Andrea T. Da Poian, Christine Cruz-Oliveira, Iranaia Assunção-Miranda

**Affiliations:** 1 Instituto de Microbiologia Paulo de Goes, Centro de Ciências da Saúde, Universidade Federal do Rio de Janeiro (UFRJ), Rio de Janeiro, Brazil; 2 Instituto de Bioquímica Médica Leopoldo de Meis, Centro de Ciências da Saúde, Universidade Federal do Rio de Janeiro (UFRJ), Rio de Janeiro, Brazil; 3 Instituto de Biologia, Universidade Federal do Rio de Janeiro (UFRJ), Rio de Janeiro, Brazil; 4 NUMPEX - Núcleo Multidisciplinar de Pesquisas, Polo Avançado de Xerém, Universidade Federal do Rio de Janeiro (UFRJ), Duque de Caxias, RJ Brazil; 5 000 0004 0620 4442 Seção de Arbovirologia e Febres Hemorrágicas, Instituto Evandro Chagas (IEC), Ananindeua, PA Brazil; 6 Departamento de Morfologia e Ciências Fisiológicas, Centro de Ciências Biológicas e da Saúde, Universidade do Estado do Pará (UEPA), Belém, PA Brazil

## Abstract

The global situation of diseases transmitted by arthropod-borne viruses such as Dengue (DENV), Yellow Fever (YFV), Chikungunya (CHIKV) and Zika (ZIKV) viruses is alarming and treatment of human infection by these arboviruses faces several challenges. The discovery of broad-spectrum antiviral molecules, able to inactivate different groups of viruses, is an interesting approach. The viral envelope is a common structure among arboviruses, being a potential target for antivirals. Porphyrins are amphipathic molecules able to interact with membranes and absorb light, being widely used in photodynamic therapy. Previously, we showed that heme, Co-protoporphyrin IX (CoPPIX) and Sn-protoporphyrin IX (SnPPIX) directly inactivate DENV and YFV infectious particles. Here we demonstrate that the antiviral activity of these porphyrins can be broadened to CHIKV, ZIKV, Mayaro virus, Sindbis virus and Vesicular Stomatitis virus. Porphyrin treatment causes viral envelope protein loss, affecting viral morphology, adsorption and entry into target cells. Also, light-stimulation enhanced the SnPPIX activity against all tested arboviruses. In summary, CoPPIX and SnPPIX were shown to be efficient broad-spectrum compounds to inactivate medically and veterinary important viruses.

## Introduction

Arthropod-borne viruses (arboviruses) are the etiologic agents of many incapacitating diseases that can progress to severe and lethal forms, affecting the human population worldwide^1,2^ and are therefore considered a global health problem by World Health Organization (WHO). Different groups of viruses are included in this classification, such as Dengue virus (DENV), Yellow Fever virus (YFV), Mayaro virus (MAYV), Sindbis virus (SINV), West Nile virus (WNV), Chikungunya virus (CHIKV), Zika virus (ZIKV) and Vesicular Stomatitis virus (VSV), a virus of veterinary relevance. The geographic distribution of the arboviruses has expanded throughout the last years, and their introduction into new areas resulted in great increase in the number of people at risk^1–3^.

The current situation of arboviruses-transmitted diseases is alarming. It is estimated that approximately 96 million people develop the clinical form of DENV infection annually^4^. Also, the presence of CHIKV has been confirmed in Asia, Africa, Europe and more recently in the Americas, where local transmission has been confirmed in over 43 countries and territories after virus introduction in 2014^5^. Despite the low mortality rate associated with CHIKV infection, approximately 80% of infected patients develop chronic debilitating joint pain, resulting in a high economic impact for the health system^6,7^. Another example of the rapid change in the arboviruses’ geographic distribution is the ZIKV introduction in the American continent. Between 2015 and 2016, several cases of ZIKV infection were notified in 55 new countries and territories^8^. When symptomatic, ZIKV infection is usually characterized as a mild febrile condition in adults, but in pregnant women it was associated with fetal malformation and newborn neurological complications^9,10^. In addition, ZIKV infection has been associated with neurological complications in adults, such as Guillian-Barré syndrome^11^. Together, these observations highlight the need of new approaches in arbovirus control and disease treatment.

Besides the risk of disease emergence and re-emergence, treatment of the different arboviruses faces several challenges, including the lack of specific drugs, co-infection of different co-circulating arboviruses and difficulties in early clinical diagnosis due to the overlap of symptoms^12–15^. Therefore, treatment with molecules showing broad-spectrum antiviral activity is an interesting approach. Although the replication strategies vary among the different arboviruses, they are all enveloped viruses. Thus, compounds that target viral envelope are promising candidates for the development of broad-spectrum antiviral drugs. Although cellular membrane is capable of self-recycling, allowing for rapid damage repair, the viral envelope does not have this ability, making them susceptible to envelope-targeting drugs^16^. Some envelope-targeting compounds have been described as promising drugs that inhibit the entry steps of viral infection cycle^17^. These molecules range from organic lipophilic to peptidic nature, which interacts with lipid components or glycoproteins from the envelope, respectively, decreasing viral infectivity *in vitro* and *in vivo*^18–25^.

Porphyrins are a large class of natural and synthetic organic heterocyclic molecules that can bear a metal atom in the center of their tetrapyrrolic structure. Some of these molecules can be photosensitized and are often used in photodynamic therapy for microorganism killing^26–29^. Additionally, antimicrobial activity of some porphyrinic compounds has also been described in the absence of light stimuli^27^. Recently we demonstrated that non-photosensitized heme, Co-protoporphyrin IX (CoPPIX) and Sn-protoporphyrin IX (SnPPIX) inactivate DENV and YFV particles^30^. The porphyrins tested had a direct effect on viral particles, inhibiting the early steps of viral infection^30^. At the present study, we tested the hypothesis that heme, CoPPIX and SnPPIX by targeting the viral envelope, are effective to inactivate different types of enveloped viruses, such as those belonging to the very diverse group of arboviruses. We performed inactivation tests in the absence or presence of light stimulation with CHIKV, ZIKV, MAYV, VSV and SINV. Our results demonstrate that porphyrins have a potential broad-spectrum activity in the treatment and control of different enveloped viruses.

## Results

### CoPPIX and SnPPIX have a broad-spectrum ability to inactivate viruses

**Figure 1:**
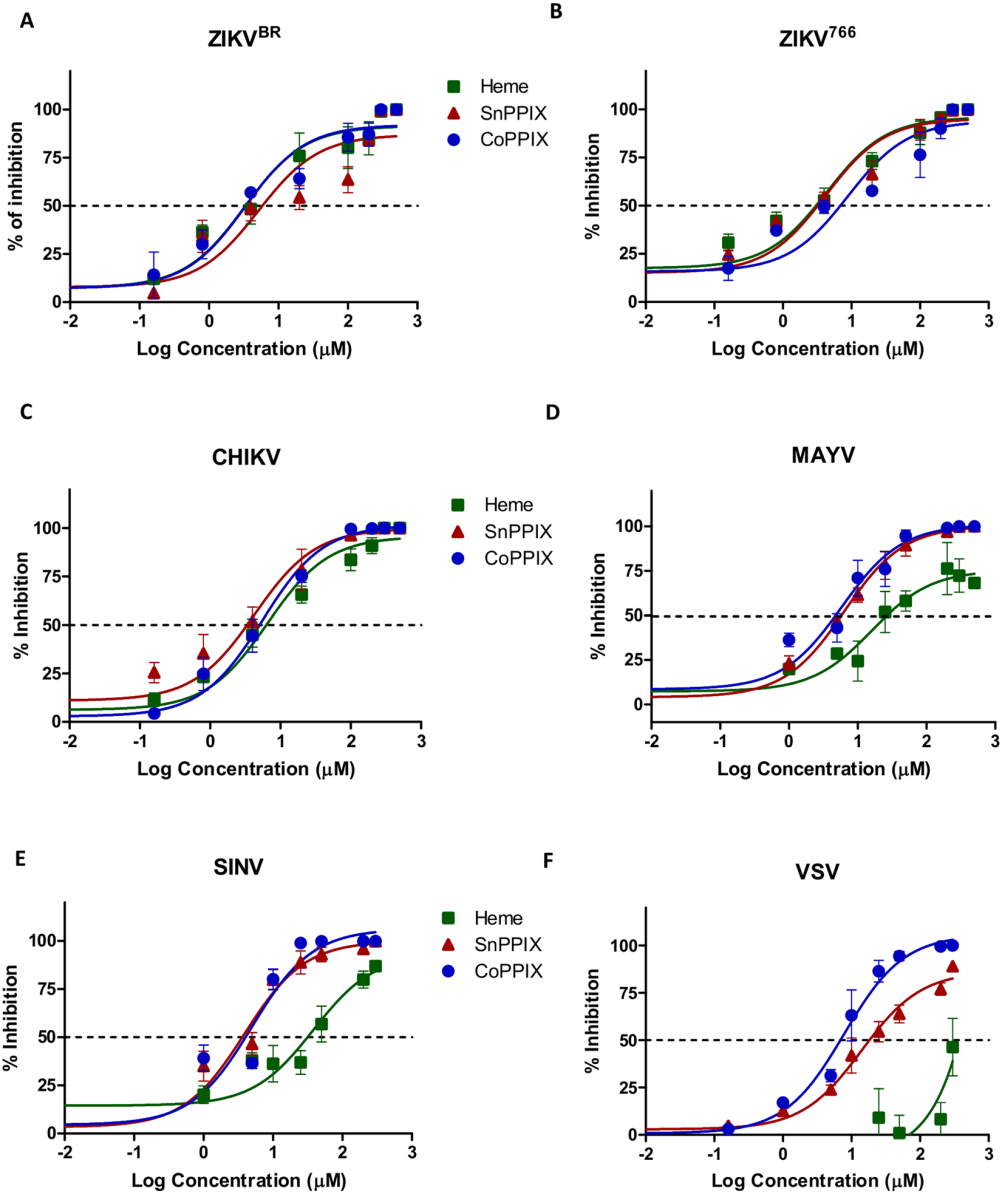
CoPPIX, SnPPIX and heme have broad-spectrum virus inactivation ability. (**A**) ZIKV^BR^, (**B**) ZIKV^766^, (**C**) CHIKV, (**D**) MAYV, (**E**) SINV and (**F**) VSV were incubated with different concentrations of heme (green square), CoPPIX (blue circles) and SnPPIX (red triangles) for 1 h, in the dark. 0.6% DMSO was used as vehicle. Afterwards, virus infectivity was assessed by plaque assay and plotted in dose-response curve with sigmoidal profile and variable slope of % of inhibition compared with untreated virus. The Graph’s dotted lines are representative of 50% inhibition in virus infectivity. Data are represented as means ± SEM of at least three independent samples.

The ability of heme, CoPPIX and SnPPIX to directly inactivate different classes of arboviruses was tested by pre-incubating CHIKV, MAYV, SINV, Brazilian and African strains of ZIKV (ZIKV^BR^ and ZIKV^766^, respectively) and VSV with different concentrations of heme, CoPPIX and SnPPIX in the dark for 1 h prior to infection. Infectious particles were quantified by plaque assay and results were plotted in % of inhibition using untreated virus as reference. Dose-response curve of CoPPIX and SnPPIX showed that the treatment significantly reduced infectivity of CHIKV, MAYV, SINV, ZIKV^BR^, ZIKV^766^ and VSV (Fig. 1), even in a lower dose than the 50% cytotoxicity concentration (CC_50_) (Table 1). At the concentration of 300 µM, SnPPIX was able to fully inactivate CHIKV (Fig. 1C), whereas CoPPIX at this concentration completely inactivated all tested arboviruses. The half maximal inhibitory concentrations (IC_50_ ± SEM) for CoPPIX and SnPPIX in the absence of light-stimuli were very similar and at micromolar range (Table 1). Heme was especially successful in inactivating ZIKV^BR^ and ZIKV^766^, also with a micromolar range IC_50_ (Table 1), but failed to inactivate SINV and VSV (Fig. 1E,F), which indicates selectivity of this porphyrin to some viruses. Determined values of porphyrin’s CC_50_ and IC_50_ show that inactivation efficiency by CoPPIX and SnPPIX treatment occur in a range of concentration lower than their cytotoxicity.

**Table 1:** Porphyrins CC_50_ and IC_50_ values (µM ± standard error, SEM).

Porphyrin	CC_50_ (µM ± SEM)	IC_50_ (µM ± SEM)					
		ZIKV^BR^	ZIKV^766^	CHIKV	MAYV	SINV	VSV
		**Without Light-stimulation**					
CoPPIX	762.3 ± 2.4	2.49 ± 0.31	10.68 ± 3.02	5.01 ± 1.26	5.94 ± 2.29	4.89 ± 1.90	6.68 ± 0.67
SnPPIX	816 ± 1.27	5.98 ± 2.63	7.78 ± 1.76	4.86 ± 2.07	5.99 ± 1.71	2.44 ± 2.30	8.86 ± 1.34
Heme	525 ± 1.08	2.59 ± 1.53	6.45 ± 2.39	5.55 ± 1.44	**ND**	**ND**	**ND**
		**Under Light-stimulation**					
CoPPIX	1018 ± 3.23	5.79 ± 0.91	8.05 ± 2.42	5.29 ± 3.21	7.48 ± 2.37	4.89 ± 1.90	3.84 ± 1.14
SnPPIX	20.7 ± 4.16	0.16 ± 0.04	0.12 ± 0.07	0.26 ± 0.08	0.09 ± 0.08	0.33 ± 0.26	0.23 ± 0.04
Heme	>750	4.14 ± 1.02	5.27 ± 1.66	10.76 ± 2.31	**ND**	**ND**	**ND**

ND- Non-determined IC_50_.

**Figure 2:**
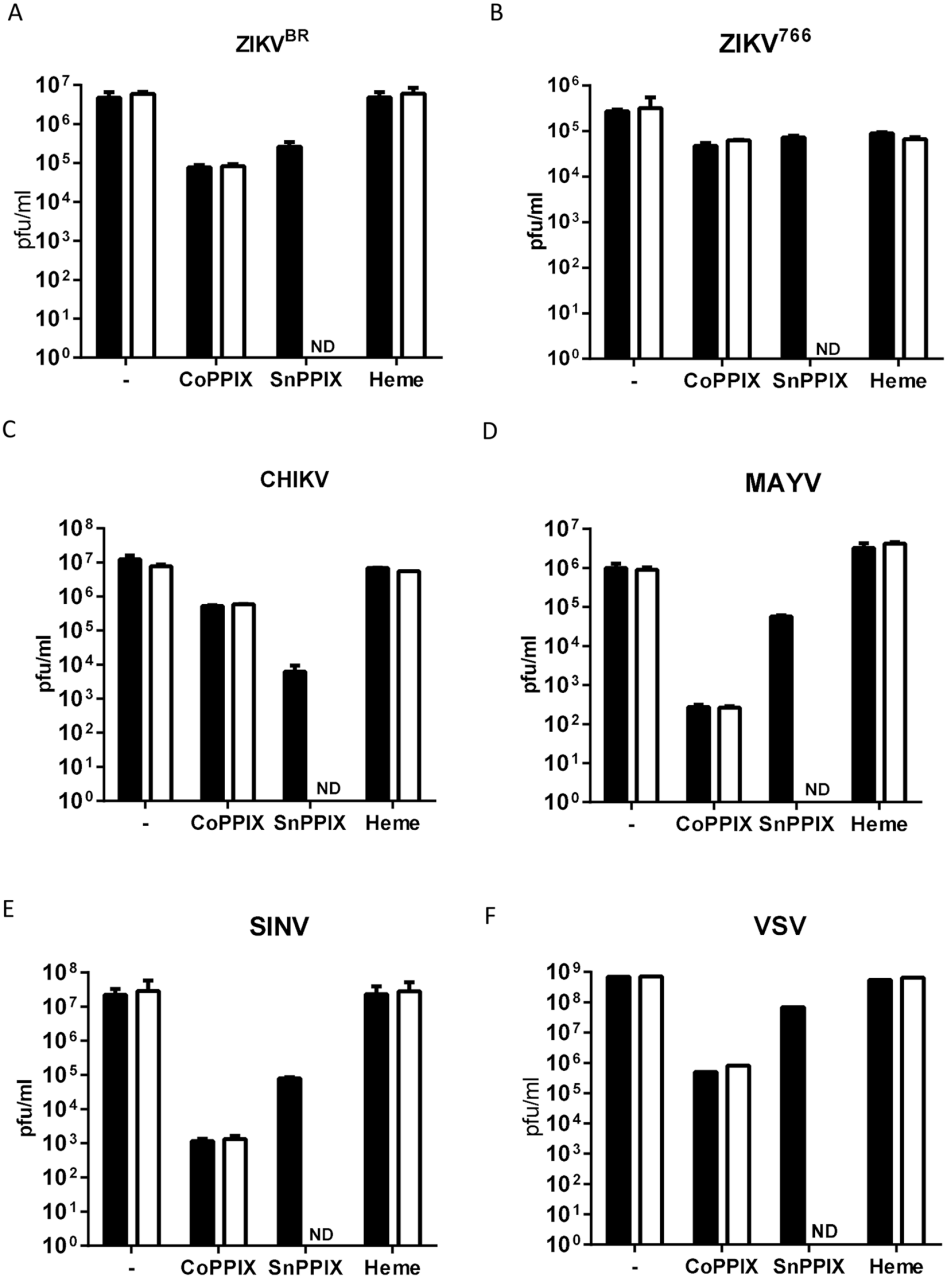
Light-stimuli enhance SnPPIX inactivation efficiency. (**A**) ZIKV^BR^, (**B**) ZIKV^766^, (**C**) CHIKV, (**D**) MAYV, (**E**) SINV and (**F**) VSV were treated with 200 µM CoPPIX, SnPPIX or heme under light-stimuli (white bars) or without light-stimuli (black bars). 0.6% DMSO was used as vehicle. Afterwards, virus infectivity was assessed by plaque assay. ND indicates samples with non-detected pfu. Data are represented as means ± SEM of at least three independent samples.

Since some porphyrins can be photosensitized, we investigated whether exposure to light would enhance the effect of heme, CoPPIX and SnPPIX. To test the light exposure effect, arboviruses were treated with 200 µM of porphyrin and exposed, or not, to 500 lux (lx) for 10 min and then incubated for 1 h at 37 °C in the dark. No differences in virus titer were found between light-exposed and non-exposed CoPPIX- or heme-treated samples, indicating that their effects are independent of light stimulation (Fig. 2A–F). In contrast, SnPPIX was much more efficient in reducing the number of infectious particles when photo-stimulated when compared to the non-light-stimulated condition (Fig. 2A–F). Light-stimulated SnPPIX was able to completely inactivate all tested arboviruses at 200 µM, which was not observed in absence of light-stimulation even with 300 µM (Fig. 1). Comparison of IC_50_ of SnPPIX obtained in stimulated and non-stimulated conditions shows that light exposure promotes an enhancement of inactivating activity of about 37-fold to ZIKV^BR^, 64-fold to ZIKV^766^, 18-fold to CHIKV, 66-fold to MAYV, 25-fold to SINV and 38-fold to VSV (Table 1).

In order to confirm inactivation by porphyrins, viral particles were incubated with 300 µM CoPPIX, SnPPIX or 10 µM SnPPIX under light stimuli and their ability to induce cell death and synthesize viral proteins in susceptible cells were tested (Figs S1 and S2, respectively). MTT assay results showed that treated viruses had a significant loss in the ability to induce cell death when compared with untreated viruses (Fig. S1A–F). Non-photo-stimulated SnPPIX failed only to protect against SINV infection but, under light stimuli, it was possible to observe cell protection even with a lower dose of SnPPIX (Fig. S1E). Heme was more efficient against ZIKV^BR^ and ZIKV^766,^ which is consistent with our previous results with other members of the Flaviviridae family^30^. Cell viability was unaffected by porphyrin incubation in non-infected conditions. In addition, viral envelope proteins were not detected by immunofluorescence microscopy in cells infected with arboviruses pre-treated with CoPPIX or SnPPIX under light stimulation (Fig. S2). These data indicate that CoPPIX, SnPPIX and heme are efficient in inactivating *Flaviviridae*, *Togaviridae and Rhabdoviridae* members, which demonstrates the broad-spectrum of these molecules’activity.

### Inhibition of ZIKV and CHIKV infection’s progress by treatment with SnPPIX and CoPPIX

**Figure 3:**
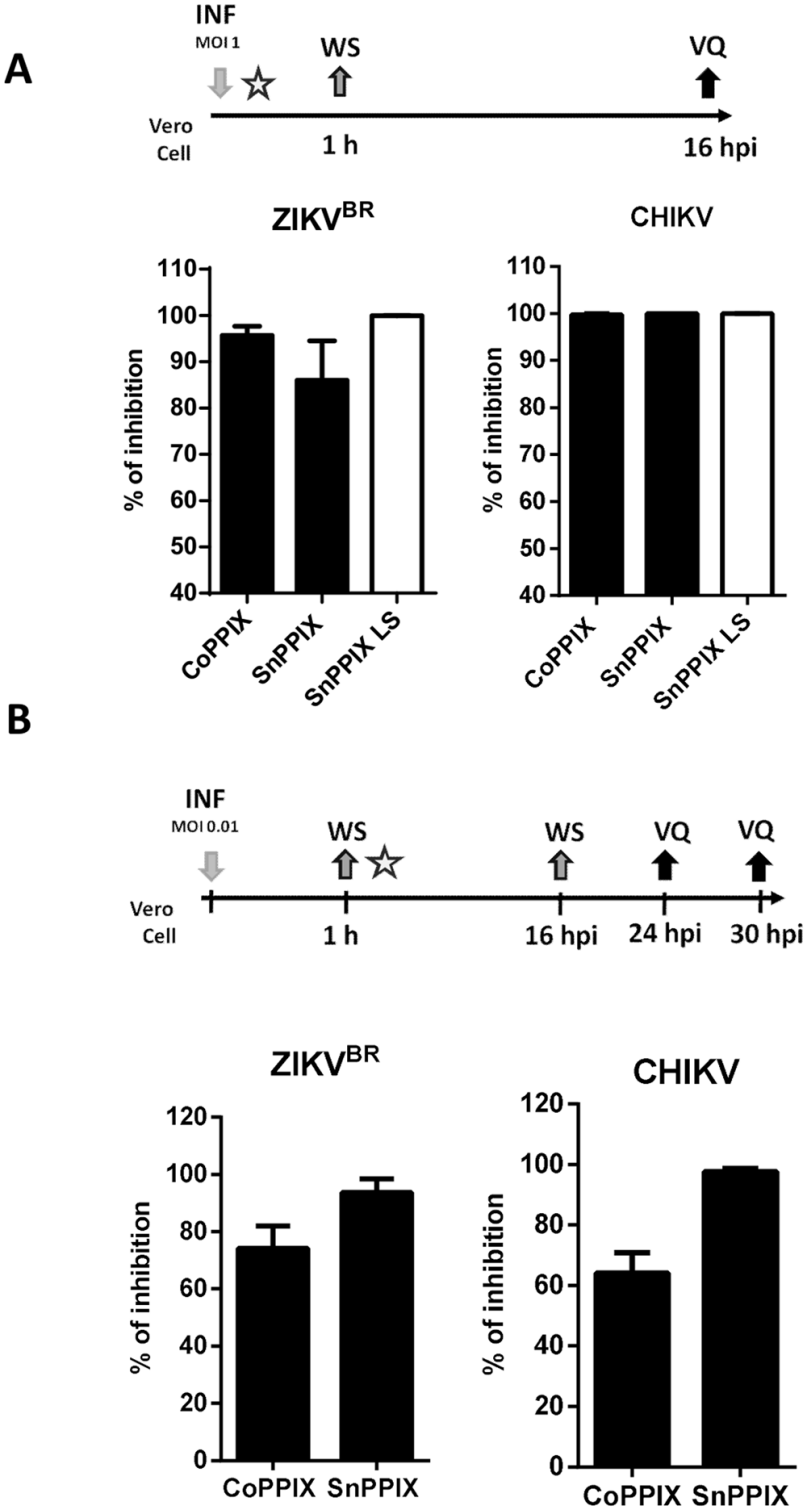
Inhibition of ZIKV and CHIKV infection progression in CoPPIX and SnPPIX-treated cells. (**A**) Cells were treated with CoPPIX and SnPPIX (100 µM) concomitantly with ZIKV^BR^ or CHIKV infection of Vero cells (MOI 1), then porphyrin and cells were cultured for 16 hpi until virus quantification. (**B**) Vero cells were infected with low MOI (0.01) and treated with CoPPIX and SnPPIX (100 µM) for 16 h. After porphyrin removal, virus quantification was performed 30 hpi for ZIKV^BR^ or 24 hpi for CHIKV. % of inhibition was calculated using untreated virus as reference. Timeline schematic representations of assay steps are shown above graphs. Porphyrin treatment is represented by the star; Infection (INF); wash with PBS (WS); Viral quantification (VQ). Data are represented as means ± SEM of at least three independent experiments.

Since we demonstrated that CoPPIX and SnPPIX are able to directly inactivate different arboviruses, we have decided to investigate whether these porphyrins would be able to control infection progression in cells. For this, we treated Vero cells with CoPPIX (100 µM) and SnPPIX (100 µM or 10 µM when LS) at the moment of ZIKV^BR^ or CHIKV infection with a MOI of 1, in order to prevent viral infection (Fig. 3A). Treatment with CoPPIX promoted an inhibition of infection in the order of 95% and 99% for ZIKV and CHIKV, respectively. SnPPIX’s efficiency was more pronounced when LS, having ZIKV and CHIKV been almost completely inactivated. We have also tested the efficiency of CoPPIX and SnPPIX, treating cells after infection in order to affect virus released from infected cells. For this, we have infected Vero cells with low MOI (0, 01) and cells were then treated with CoPPIX (100 µM) and SnPPIX (100 µM) for 16 hpi (Fig. 3B). Virus quantification in the culture medium, 1 replication cycle after porphyrin removal from the culture, demonstrated that porphyrin treatment was able to inhibit ZIKV and CHIKV infection (Fig. 3B). The treatment was more efficient when performed simultaneously with the infection, where virtually all viral particles could be assessed by the porphyrins.

### CoPPIX and SnPPIX act impairing viral adsorption and entry in susceptible cells

**Figure 4:**
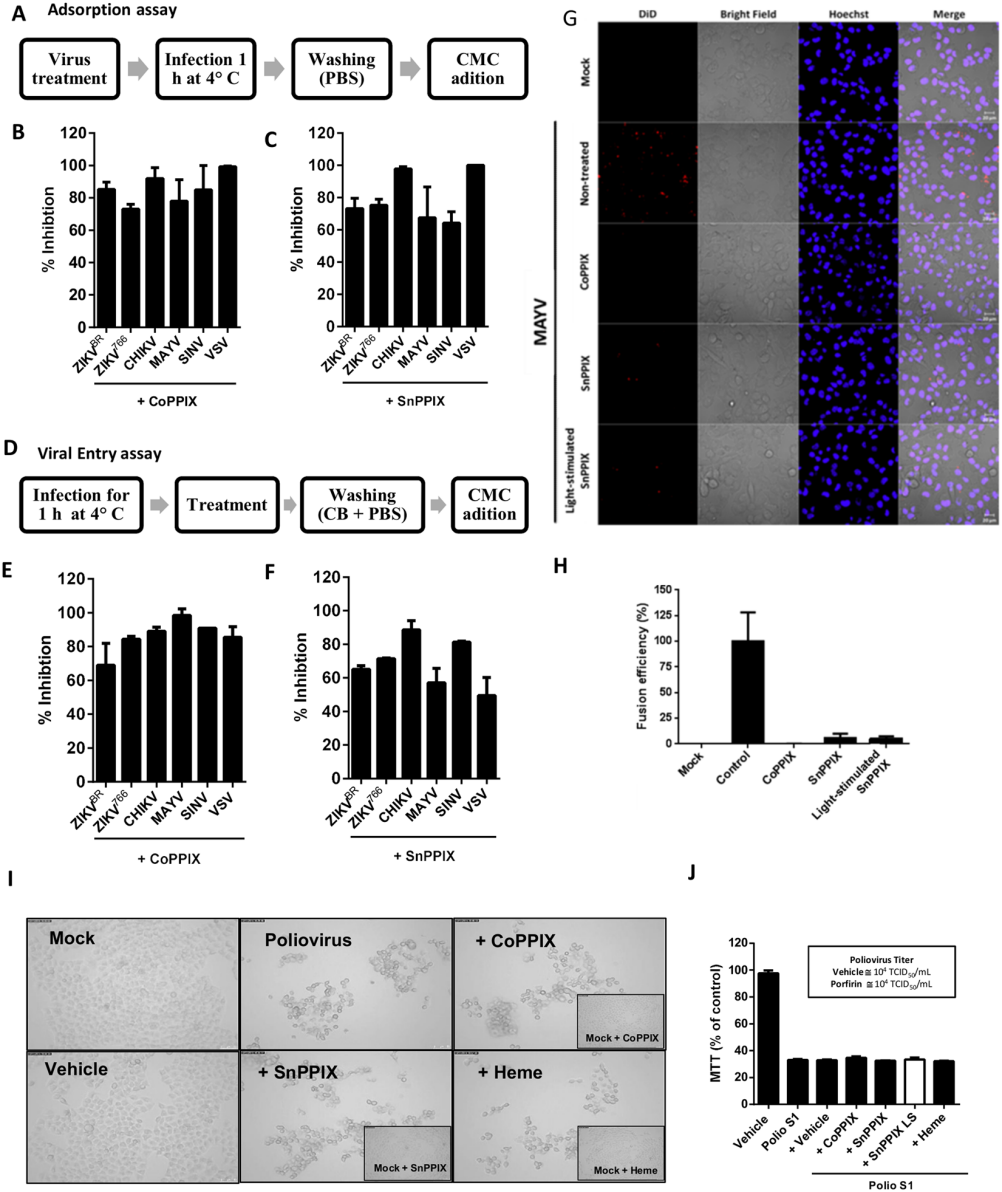
CoPPIX and SnPPIX impair viral adsorption and fusion. (**A**) Schematic representation of the adsorption assay steps. (**B**) Adsorption inhibition of 300 µM CoPPIX and (**C**) 300 µM SnPPIX-treated virus. (**D**) Schematic representation of the viral entry assay steps. (**E**) Entry inhibition percentage of 300 µM CoPPIX and (**F**) entry inhibition percentage of 300 µM SnPPIX. Inhibition was assessed in percentage comparing with untreated virus. (**G**) Representative image of fusion assay of Did-labeled MAYV treated with 300 µM CoPPIX or SnPPIX or 10 µM SnPPIX under light-stimuli obtained by fluorescence microscopy with magnification of 10x. (H) Fusion efficiency quantification from at least 10 images for each condition. (**I**) HeLa cells were infected or not with poliovirus Sabin 1 (Polio S1) pre-treated with 300 µM of CoPPIX, SnPPIX and heme. After 24 h, morphology alterations were observed by microscopy under 10x magnification. Insets shows the morphology of uninfected cells treated with porphyrin. (**J**) Quantification of HeLa viability was assessed by MTT. For SnPPIX LS treatment the dose used was 10 µM. TCID_50_ values for nontreated or porphyrin-treated poliovirus are shown in the box. Data are representative of at least 3 independent experiments or represented as means ± SEM of at least three independent experiments.

Next, we investigated if CoPPIX or SnPPIX treatments were able to inhibit the initial infection stages of the different arboviruses tested. For this purpose, we performed adsorption and entry inhibition assays according to the scheme shown in Fig. 4A,D, respectively. The effect of 300 µM CoPPIX or SnPPIX on arboviruses’ adsorption and entry steps was assessed by plaque assay. Porphyrin treatment of the different arboviruses promoted a marked reduction in virus adsorption (Fig. 4B,C) and entry (Fig. 4E,F) in BHK-21 cells. To further investigate the ability of CoPPIX and SnPPIX to inhibit arbovirus entry, we performed an assay using DiD-stained MAYV, which only emits fluorescence when the viral envelope fuses with the endosomal membrane during virus endocytosis. The images obtained by fluorescence microscopy showed that after treatment with 300 µM CoPPIX, 300 µM SnPPIX or10 µM SnPPIX with light stimuli, MAYV was not able to efficiently fuse to the endosomal membrane (Fig. 4G,H). Altogether, these results indicate that the tested porphyrins act on the viral envelope, impairing virus ability to adsorb and enter target cells.

### Non-enveloped poliovirus infection is not affected by CoPPIX or SnPPIX treatment

To evaluate the requirement of lipid envelope for viral inactivation promoted by CoPPIX, SnPPIX and heme, we tested the effect of these prophyrins on the infectivity of a non-enveloped virus, the Sabin 1 poliovirus. For this, we infected HeLa cells with poliovirus treated or not with 300 µM CoPPIX, SnPPIX or heme and observed the cytophatic effect induced by the virus. Changes in cell morphology and death induced by porphyrin-treated poliovirus were similar to that induced by untreated virus (Fig. 4I). Consistently with morphological observations, MTT assay showed that treated and untreated poliovirus equally reduced viability of HeLa cells and viral titer was also unaltered between the two groups, even when using SnPPIX LS (Fig. 4J). Inefficiency of porphyrins in inactivating a non-enveloped virus further supports the idea that they target the viral envelope.

### CoPPIX and SnPPIX alter the integrity of arboviruses envelopes preserving antigenicity

**Figure 5:**
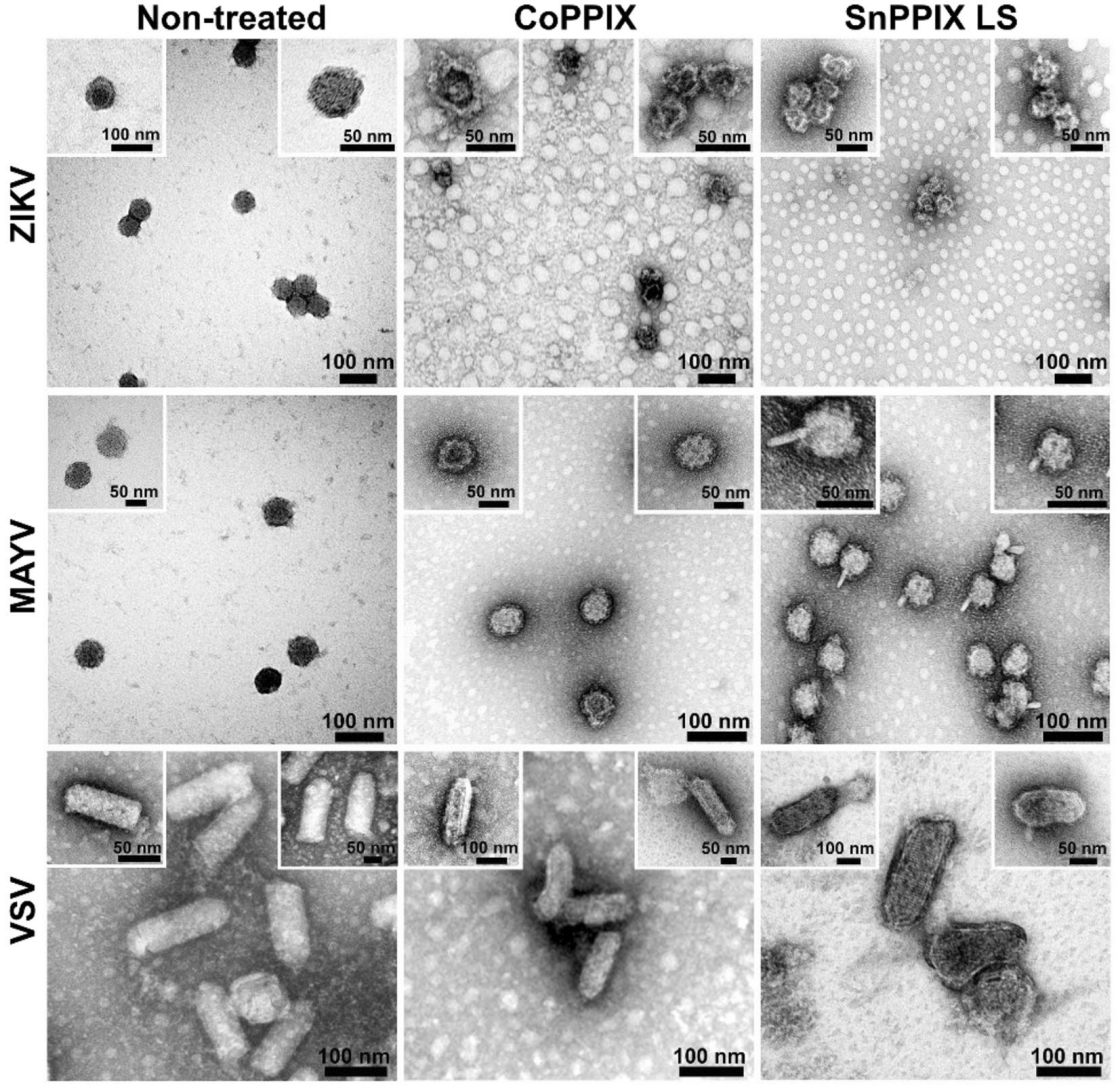
CoPPIX and SnPPIX alter the integrity of arbovirus envelope. Transmission electron microscopy images of non-treated, 300 µM CoPPIX-treated or 10 µM SnPPIX-treated under light-stimuli (LS) ZIKV^BR^, MAYV and VSV. The images were captured with magnifications of 160,000 and 305,000x.

Analysis of viral particles by transmission electron microscopy shows that the viral envelope structure was affected after treatment with CoPPIX and light-stimulated SnPPIX (Fig. 5). CoPPIX-treated ZIKV and MAYV particles exhibit irregularities in particle structure resulting in symmetry loss. In addition, the images show an apparent curvature on the surface of ZIKV particles. The particle structure irregularities were also observed after treatment with light stimulated-SnPPIX (Fig. 5), with a tendency of viral particle aggregation as well as the appearance of a projection on the surface of treated-MAYV particles.

**Figure 6:**
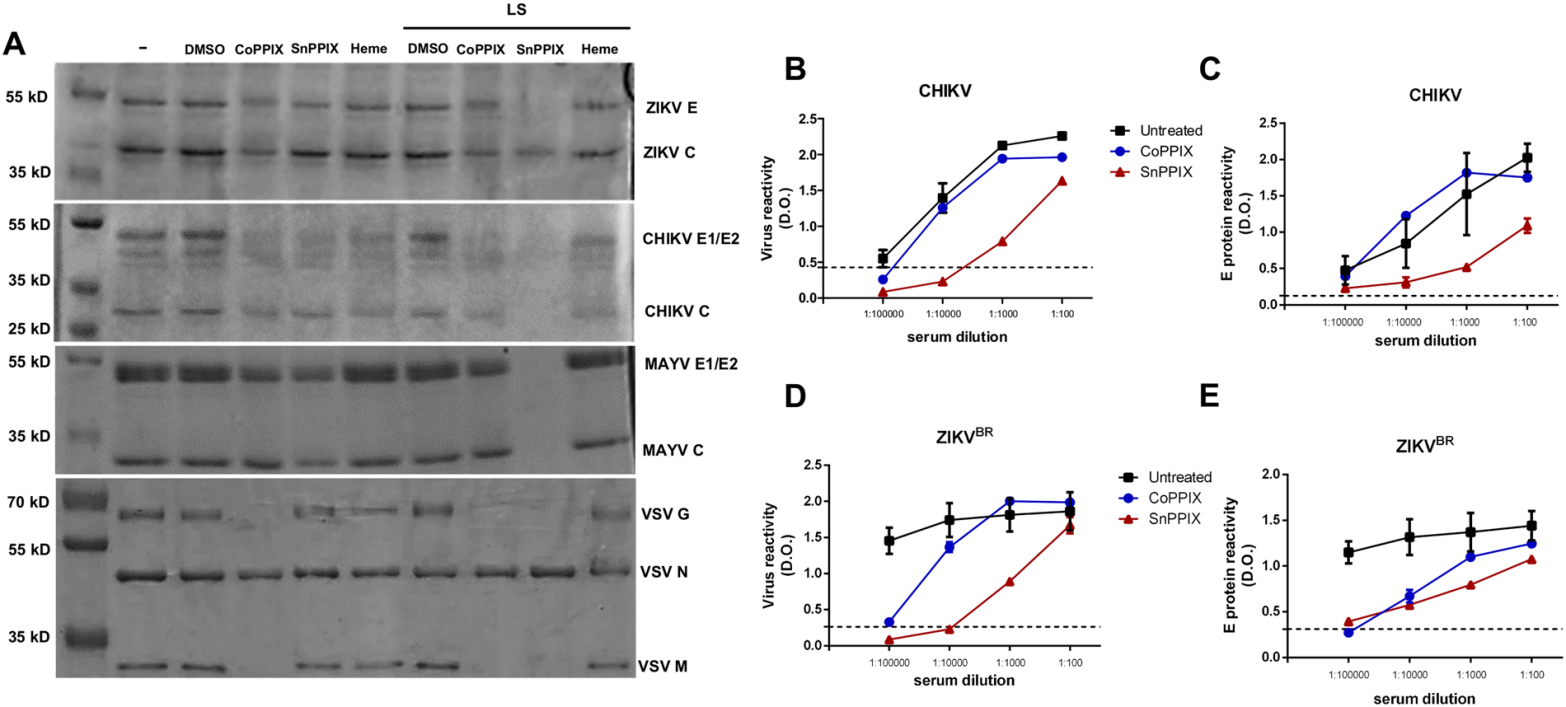
CoPPIX and SnPPIX disrupt viral envelope protein integrity but preserves virus antigenicity. (**A**) Viral protein band pattern of ZIKV^BR^, CHIKV, MAYV and VSV treated with 300 µM CoPPIX, SnPPIX or heme with or without light stimuli (LS). Viral proteins separation by electrophoresis were performed in independent gels which were cropped for visualization of specific bands. Viral proteins are indicated for each virus: ZIKV envelope (**E**) and capsid (**C**) proteins; CHIKV and MAYV envelope 1/2 (E1/E2) and capsid (**C**) proteins; VSV glycoprotein (G), nucleocapsid (N) and membrane (M) proteins. SDS-PAGE gels are representative of at least 3 independent experiments. Full-length gels are presented in Supplementary Figure 3. (**B**–**E**) Antigenicity of nontreated or porphyrin-treated virus was evaluated by ELISA using serial dilution of anti-CHIKV and anti-ZIKV positive human serum. (**B**,**D**) Plate was coated with treated or untreated virus. (**C**,**E**) Plate was coated with antibody against E protein of CHIKV or ZIKV and then treated or untreated viruses were added. Data are represented as means ± SEM of two independent experiments.

In order to determine the effect of CoPPIX, SnPPIX or heme on viral envelope proteins, we analyzed porphyrin-treated and untreated purified viruses by SDS-PAGE. Protein band analysis showed that treatment with CoPPIX reduced E/G protein band intensity of ZIKV, CHIKV, MAYV and VSV (Fig. 6A). This reduction was also observed in M protein band intensity in CoPPIX-treated VSV as well in C protein band intensity of ZIKV and CHIKV. As expected, light stimuli enhanced the effects of SnPPIX. Treatment with SnPPIX following light stimuli resulted in intensity decrease even in C protein band of ZIKV and MAYV and M protein band of VSV. In agreement with the infectivity results, treatment with heme also promotes significant alteration in protein band pattern, being the effects more pronounced on ZIKV and CHIKV samples (Fig. 6A). These results indicate that CoPPIX, SnPPIX and heme treatments affect viral envelope protein content.

Since porphyrins disrupt the viral envelope integrity, we decided to evaluate if inactivated viral particles would lose their antigenicity. In order to do this, we tested the reactivity of whole-ZIKV and CHIKV coated plates with serial dilutions of anti-ZIKV or anti-CHIKV positive human serum by ELISA, after treatment with 300 µM of CoPPIX and SnPPIX (Fig. 6B,D). With this analysis, we could observe that CoPPIX treatment almost did not interfere with serum reactivity against CHIKV and ZIKV when compared to the reactivity profile of untreated virus. Although treatment with SnPPIX decreases serum reactivity, treated ZIKV and CHIKV are still recognized in lower serum dilution, indicating that virus preserves certain antigenicity. In order to evaluate preservation of specific E protein antigenicity, we performed an ELISA, coating plates with ZIKV or CHIKV anti-E protein antibody before addition of untreated or treated virus. Following, we tested the reactivity for positive human serum serial dilutions (Fig. 6C,E). We found that treated ZIKV and CHIKV could still be captured by E specific antibodies, reacting positively with serum, even though to a lesser extent when compared to untreated viruses. As observed in the test of total viral reactivity, CoPPIX treatment promoted less interference in E protein reactivity, when compared with SnPPIX. Taken together, these results show that even though treated virus lost adsorption and fusogenic ability in cells, its antigenicity is partially preserved.

## Discussion

The difficulty in containing arboviruses’ global expansion represents an important public health problem. Only in the American continent, several DENV, YFV, CHIKV and ZIKV outbreaks were reported in the last years. MAYV is endemic in Central and South America, mainly circulating in forest regions. Reported cases of MAYV fever are very restrict but do not correspond to the real circulation of this virus^31^. In this context, the possibility of treating these different arboviruses with the same group of compounds represents an advantageous strategy that should be encouraged. Here we demonstrate that treatment of different viruses with heme, CoPPIX and SnPPIX affected the integrity of the viral envelope proteins, resulting in the inability of these viruses to adsorb and enter target cells. Although restricted to enveloped viruses, CoPPIX, SnPPIX and heme have broad direct-acting inactivating activity against several arboviruses, including flaviviruses, alphaviruses and vesiculoviruses of medical and veterinary importance.

CoPPIX, SnPPIX and heme are hydrophobic molecules that may interact with and affect viral envelope lipids. It was previously demonstrated that some metalloporphyrins, including CoPPIX, intercalate the liposome bilayer, leading to lipid disorder and liposome fluidity alteration^32^. Furthermore, protoporphyrin IX (PPIX), Zn-protoporphyn IX (ZnPPIX) and mesoporphyrin (MPIX) were shown to interact with liposomes and the lipids within the viral envelope, altering envelope membrane ordering^33^. Although the porphyrins tested in the present work are very similar in structure, differing basically on their central metallic atom, there is a difference in their specificity against the different arboviruses. Indeed, heme was only effective in inactivating flaviviruses, while CoPPIX was efficient against all tested arboviruses (Table 1). This difference might be determined by envelope organization and composition, which may interfere in the fitness of their interaction. Alphaviruses, for example, present a highly packed lipid envelope with high density of viral glycoproteins, features that are crucial for infectivity^34^. CoPPIX may have a greater interaction capacity with the envelope, allowing an efficient interaction regardless of the degree of lipid exposure. Thus, porphyrin may interact and inactivate most enveloped viruses with different inactivation efficiencies according to viral particle’s envelope packaging and could be successful in inactivating a broad variety of enveloped viruses.

The ability of CoPPIX and SnPPIX to damage the viral envelope occurs even in the absence of light stimuli, but SnPPIX’s activity is markedly enhanced with an exposition to 500 lx. Photoactivation of some porphyrinic compounds generates reactive oxygen species (ROS), such as singlet oxygen, inside the envelope environment^27,29^. Light-dependent ROS generation could lead to alterations in viral protein integrity observed in light stimulated SnPPIX-treated viruses. In fact, PPIX, ZnPPIX and MPIX virucidal activity under light stimuli is related to singlet oxygen generation and protein modification^33^. Furthermore, inactivation tests using light stimuli were carried out with a single luminous intensity value. An increase in luminous intensity might promote a greater SnPPIX virucidal efficiency, further reducing the IC_50_ for different enveloped viruses.

Adsorption and entry of ZIKV, CHIKV, MAYV, SINV and VSV were affected by CoPPIX and SnPPIX treatment. Electron microscopy images showed that treated viral particles exhibit significant deformations on their surfaces. CoPPIX-treated ZIKV and SnPPIX-treated MAYV particles presented surface depressions and projections, respectively. Furthermore, the reduction of some virus protein bands in SDS-PAGE could also represent some major structural modifications of the viral envelope, such as protein loss. Moreover, it is possible that the lipid bilayer is also being affected by CoPPIX and SnPPIX treatment, altering envelope curvature. Although it was not possible to determine the exact modifications on viral envelope proteins, they should impair the initial virus-cell interactions, inhibiting cell entry. This way, treated viruses become unable to infect susceptible cells.

Despite all modifications induced by CoPPIX and SnPPIX, ELISA data indicates that inactivated virus can still be recognized by antibodies present in positive human serum, even though at a lower level compared to intact virus. Usually the glycoproteins of enveloped viruses are the ones responsible for antigenicity. Thus, our results suggest that even though envelope glycoproteins were functionally impaired by CoPPIX and SnPPIX, they keep some structural information. Preservation of virus antigenicity is an important feature, since even inactivated, immune response for the virus would be inducible and be safety used as possible vaccine.

Other compounds targeting viral envelope have been tested for virus inactivation, supporting this strategy as promising in the development of broad-spectrum compounds against enveloped viruses. Mastoparan, a natural host defense peptide, and its derivative, MP7-NH_2_, are able to alter virus envelope by interacting with lipid components^22,24^. LJ001 compound, a lipophilic thiazolidine, and its soluble derivatives, JL118 and JL122, have been characterized as membrane fusion inhibitors^21^. These molecules interact with the viral envelope, generating singlet oxygen upon luminous stimuli, which is the mechanism by which they promote fusion inhibition^35^. Porphyrins present similar lipid interaction properties and are also able to induce singlet oxygen formation. They are therefore also candidates to be used against enveloped virus infection.

The safety, as well as the therapeutic efficiency of CoPPIX and SnPPIX’s use in arbovirus infections, need to be investigated in *in vivo* assays. These compounds’ ability to inhibit infection progression in cell culture, on the other hand, even in treatment after infection and using lower doses than the CC_50_, indicates the potential of this approach. Interestingly, SnPPIX has already been used to control hyperbilirubinemia in rats and humans due to its heme oxigenase enzyme (HO-1) inhibitory activity^36,37^. In addition to that, CoPPIX has been used as an inducer of HO-1 in different experimental models *in vivo*, including for roles such as tissue protection and control of parasitic infection^38,39^. HO-1 catalyzes heme degradation, generating biliverdin and carbon monoxide (antioxidant products). Enzyme activity was associated with cell protection and reduction of inflammation in infectious and non-infectious diseases^38–40^. Although not directly related to SnPPIX and CoPPIX inactivating activity, its modulatory action on HO-1 suggests that it has clinical potential and could be used for treatment of viral diseases. Since HO-1 can exert a significant antiviral activity against different viruses, such as hepatitis C and B viruses, enterovirus 71, influenza virus, respiratory syncytial virus, Ebola virus and DENV^41^, treatment with CoPPIX could promote cellular and tissue protection, additionally to its arboviral inactivation activity. However, systemic use of CoPPIX and SnPPIX needs further investigation^36,37^.

Altogether our results support that CoPPIX and SnPPIX are promising candidates to be used as broad-spectrum compounds against enveloped viruses. Furthermore, it is possible to modify their structures in order to achieve greater efficiency and bioavailability. Another possibility is to use CoPPIX and SnPPIX for topical treatment of viral diseases such as cutaneous herpes ulceration. Also, porphyrins could be used as a component of virucidal ointments for sexually transmitted viruses, a route already demonstrated for ZIKV infection^42^. Considering the high prevalence of sexually transmitted viral infections, the development of new alternatives for topically applied treatment has been recommended^43^. The enhancement of SnPPIX’s efficiency upon light stimulation would significantly reduce the dose necessary for viral inactivation in topical use. Finally, CoPPIX and SnPPIX could also be used for arbovirus inactivation in vaccines since viral particles’ antigenicity is partially preserved after treatment. All these possibilities remain open, requiring further investigation of the potential use of these molecules as broad-spectrum compounds against viruses.

## Methods

### Cells

Baby hamster kidney fibroblasts (BHK-21) were cultured in MEM-α, African green monkey fibroblasts (Vero) and human cervix tumor epithelial cells (HeLa) were both cultured in DMEM and maintained at 37 °C in 5% CO_2_ atmosphere. *Aedes albopictus* cells (C6/36) were grown in L-15 medium supplemented with 0.3% tryptose phosphate broth, 0.75 g/L sodium bicarbonate, 1.4 mM glutamine and 1% nonessential amino acids at 28 °C. All cells were supplemented with 10% heat-inactivated fetal bovine serum (FBS).

### Viruses

MAYV, SINV and VSV were propagated in BHK-21 cells, ZIKV^766^ in Vero cells and CHIKV and ZIKV^BR^ in C6/36 cells using a multiplicity of infection (MOI) of 0.01. After infection, cells were cultured for 20, 24, 30, 48 and 72 h in VSV, SINV, MAYV and ZIKV^766^ infection, respectively and 7 days in ZIKV^BR^. The medium collected from each infection was centrifuged at 700 g to remove cellular *debris*, stored in aliquots at −80 °C and titrated by plaque assay. ZIKV^BR^ was isolated from a febrile case in the state of Pernambuco, Brazil (gene bank ref. number KX197192) and the stock of Sabin 1 vaccine strain of poliovirus was kindly provided by Laboratório de Enterovirus from Instituto Oswaldo Cruz and used for observation of cytopathic effect, cell viability and TCID_50_ quantification on HeLa cells. Purified ZIKV^BR^, CHIKV, MAYV and VSV were used in SDS-PAGE electrophoresis and morphology studies by transmission electron microscopy were obtained following a second step after propagation. Approximately 300 mL of medium from infected cell cultures were harvested and then centrifuged at 700 g for 10 minutes at 4 °C. Next, the supernatant was centrifuged at 150,000 g for 2.5 h at 4 °C using a 45Ti rotor (Beckman). The supernatant was discarded and 200 µL of 3E buffer (0.12 M Tris-Base, 0.06 M sodium acetate and 3 mM EDTA, pH 7.4) was added to the pellet and incubated overnight at 4 °C to dilute the virus pellet. The pellet was then ultra-centrifuged in a discontinuous 10–60% (w/v) sucrose gradient at 116,000 g (ZIKV^BR^, CHIKV and MAYV) or 180,000 g (VSV) for 1.5 h at 4 °C, using a SW41 rotor (Beckman). On each procedure, the opaque layer corresponding to the purified viral particles was collected. The viruses were stored at −80 °C and titrated by plaque assay. All the procedures and experiments were performed in facilities according to biosafety level of all studied viruses (BSL-2: ZIKV, MAYV, SINV, VSV and Poliovirus; BSL-3: CHIKV).

### Porphyrins

CoPPIX and SnPPIX (Frontier Scientific Inc.) were prepared in stocks of 50 mM in dimethyl sulfoxide (DMSO; Sigma Co.) and stored at −20 °C. On each used concentration, the final concentration of DMSO never exceeded 0.6%. All these steps were done in the absence of light. Heme (Frontier Scientific Inc.) was prepared before each experiment in a 5 mM stock. Heme was first solubilized in 100 mM NaOH and then in phosphate-buffered saline (PBS; 137 mM NaCl, 2.7 mM KCl, 10 mM Na_2_HPO_4_ and 1.8 mM KH_2_HPO_4_, pH 7.4).

### Virus treatment and quantification

To determine the effect of heme, CoPPIX and SnPPIX on arboviruses, 10^7^ plaque forming units (PFU) of virus were pre-treated with different porphyrin concentrations ranging from 0.16 to 500 µM for 1 h at 37 °C, in the dark. For light-stimulated conditions, a previous step of light exposition was added, using a 30 W fluorescent lamp of luminous emittance of 500 lx, for 10 minutes, before incubation for 1 h at 37 °C in the dark. After treatment, viral samples were serially diluted (10-fold) for titration and incubated for 1 h at 37 °C and 5% CO_2_ with BHK-21 cells for MAYV, CHIKV and VSV samples or Vero cells for ZIKV^BR^ and ZIKV^766^ samples. After infection, the medium was removed and 1 mL of MEM-α for BHK-21 cells or DMEM for Vero cells containing 1% FBS, 100 u/mL penicillin, 100 µg/mL streptomycin (LGC Biotecnologia) and 1.5% carboxymethylcellulose (CMC; Sigma Co.) was added to each well. The plates were incubated at 37 °C and 5% CO_2_. After 2 days (for MAYV, SINV and CHIKV), 20 hours (for VSV) or 5 days (for ZIKV^BR^ and ZIKV^766^), cells were fixed by adding 1 mL of 4% formaldehyde for 30 min. Each plate was washed and stained with a crystal violet solution (1% crystal violet, 20% ethanol). The number of plaques on each well was counted and multiplied according to the well dilution in order to achieve the number of pfu per mL. The half maximum inhibitory concentration (IC_50_) was determined by nonlinear regression with sigmoidal profile and variable slope using the software Graphpad Prism (version 6.0) only on treatments which achieved total inhibition. Poliovirus was quantified by TCID_50_. For this, HeLa cells were infected with serial dilutions of poliovirus (10-fold) pre-treated or not with CoPPIX, SnPPIX and heme (300 µM) for 1 h in the dark. After infection the medium was replaced by DMEM high glucose with 2% FBS and presence of cytopathic effect was observed at 20 hours post infection (hpi). The 50% cell culture infectious dose was determined as described by Reed and Muench method^44^.

### Determination of cytotoxic concentration (CC_50_)

The cytotoxic concentration of porphyrins was determined by treating vero cells with 15.62 µM to 1 mM Heme, CoPPIX or SnPPIX for 1 hour at 37 °C. In light-stimulated conditions, a previous step of light exposure for 10 minutes was included. After incubation, cells were washed with PBS and cultured for 24 h in DMEM high glucose with 5% FBS. Cell viability was assessed by MTT reduction assay. Briefly, cells were incubated for1 h with 0.5 mg/mL of 3-(4,5-dimethylthiazol-2-yl)-2, 5-diphenyltetrazolium bromide (MTT; Life Technologies) at 37 °C. Next, the solution was removed and precipitated Formazan was diluted in isopropyl alcohol with 40 mM HCl. The solution was read on a spectrophotometer at 492 nm and 620 nm. Untreated cells were used as reference of 100% viability. The cytotoxic concentration 50 (CC_50_) was determined by a nonlinear regression with sigmoidal profile and variable slope using Graphpad Prism (version 6.0) software.

### Treatment in the course of cell infection

To test porphyrins efficiency in controlling the progression of infection in cells we used two protocols: 1. CoPPIX and SnPPIX (100 µM) were added to the culture medium at the moment of ZIKV^BR^ or CHIKV infection of Vero cells (MOI 1). Afterwards, cells were washed with PBS and cultured in DMEM high glucose with 2% FBS until 16 hpi. Medium was then collected for viral quantification by plaque assay; 2. Vero cells were infected for 1 h with low MOI (0.01) and cells were then washed with PBS. The medium with CoPPIX and SnPPIX (100 µM) was added and cells were cultured for 16 h before being washed with PBS. Then, a new medium without porphyrin was added and collected after a new round of viral replication cycle, 8 more h for CHIKV (24 hpi) and 14 more h for ZIKV (30 hpi) until viral quantification by plaque assay. Experimental draft was illustrated in Fig. 3A,B.

### Cell viability assay

About 10^7^ pfu of virus were pre-treated for 1 h at 37 °C with different concentrations of CoPPIX, SnPPIX and heme. Then, BHK-21 cells (for MAYV, SINV and VSV) or Vero cells (for ZIKV^BR^, ZIKV^766^ and CHIKV) were infected with a MOI. of 0.1 with the treated viruses for 1 h at 37 °C and 5% CO_2_. At this condition, porphyrins were diluted at least 100-fold before contact with cells. Then, the medium was replaced and the plates were incubated for 20 h (for MAYV, SINV, VSV and CHIKV) or 48 h (for ZIKV^BR^ and ZIKV^766^). Cell viability was assessed by MTT reduction assay as previously described. About 10^4^ TCID_50_ infection units of poliovirus were treated in the same conditions, and cell viability was determined at 16 hpi. The viability of each group was normalized to the non-treated control group. Presence of cytopathic effect was observed using an inverted optical microscope and pictures were taken under a bright field with magnifications of 20 and 40x.

### Fluorescence microscopy

BHK-21 cells (in MAYV, SINV and VSV assays) or Vero cells (in ZIKV^BR^, ZIKV^766^ and CHIKV assays) seeded in 24-well plates were infected with either CoPPIX-, SnPPIX- and heme-treated or non-treated viral particles for 1 h at 37 °C and 5% CO_2_. After that, medium was replaced by fresh medium containing 2% FBS. After 20 h (for MAYV, SINV, VSV and CHIKV) or 48 h (for ZIKV^BR^ and ZIKV^766^), the plates were fixed with 4% formaldehyde in PBS for 15 min. The production of viral proteins was assessed using either an anti-alphavirus E protein mouse monoclonal antibody (in analyses with MAYV, SINV and CHIKV) or the conditioned medium of 4G2 hybridome (anti-flavivirus mouse antibody, used to mark ZIKV envelope protein), both coupled with a goat anti-mouse Alexa Fluor 488 (Invitrogen). Images were taken on an inverted fluorescence microscope with magnifications of 10 and 40x.

### Adsorption and penetration assay

Cells were seeded in a 6-well plate. For the adsorption assays, each well was infected with 200 pfu of virus pre-treated with CoPPIX or SnPPIX for 1 h at 4 °C. Then, the wells were washed with PBS and received medium MEM-α or DMEM containing 1% FBS, 100 u/mL penicillin, 100 µg/mL streptomycin and 1.5% carboxymethylcellulose. After the time described to plaque formation, the number of pfu was determined for each condition of porphyrin-treated virus.

For the penetration assays, each well was infected with 200 pfu of virusat 4 °C in the absence of CoPPIX and SnPPIX. Then, plates were incubated at 37 °C in the presence of different treatments with CoPPIX and SnPPIX, for 0, 15, 30 and 60 min. Following, cells were washed with citrate buffer (40 mM citric acid, 135 mM NaCl and 10 mM KCl, pH 3.0) for 1 min. After washing with PBS, cells were incubated with appropriated semisolid medium (culture medium with 1.5% carboxymethylcellulose) supplemented with 1% FBS and100 u/mL penicillin, 100 µg/mL streptomycin.

### Fusion assay

2 × 10^5^ BHK-21 cells were seeded in 8-compartment plates (Nunc Lab-Tek, Thermo Scientific) 16 h before the experiment. Purified MAYV particles were incubated with 1% 1,1′-dioctadecyl-3,3,3′,3′-tetramethylindodicarbocyanine, 4-chlorobenzenesulfonate salt (DiD; Life Technologies) in PBS for 1 h. Next, the virus particles were centrifuged on a Amicon Ultra filter unit with a 100 KDa molecular weight cut-off (Millipore) and then treated with CoPPIX and SnPPIX for 1 h at 37 °C. Cells were incubated with the viruses for 30 min at 4 °C, washed with PBS to remove unbound virus particles and then incubated at 37 °C for 10 minutes to allow for virus entry. Following this incubation, cells were fixed with 3.7% paraformaldehyde. Images were taken on a LSM 510 META fluorescence microscope (Zeiss) under excitation at 633 nm and emission collected from 650 to 710 nm. The fluorescence intensity of the spots positive for DiD, indicating membrane fusion, was quantified by ImageJ software (NIH) analysis of cells infected with untreated labeled-virus in ten different images averaged and normalized by the cell number, and considered to be 100% of fusion efficiency. The same procedure was used for the images of cells infected with porphyrin-treated labeled-virus and fusion efficiency was determined relative to untreated condition.

### SDS-PAGE Eletrophoresis

Purified ZIKV^BR^, MAYV and VSV treated for 1 hour at 37 °C with CoPPIX, SnPPIX and heme were resolved by denaturing electrophoresis in a 10% polyacrylamide gel with a constant current of 20 mA. The gel was stained with Coomassie blue R-250 solution (10% acetic acid, 50% methanol and 0.25% [w/v] Coomassie Brilliant Blue) and de-stained with distilled water. Gels were cropped for visualization of specific bands. The viral proteins were identified by the expected molecular weight using protein standards of known molecular weight/mass. Full-length gels are presented in Supplementary Figure 3.

### Transmission electron microscopy

Purified ZIKV^BR^, MAYV and VSV treated or not with porphyrins were fixed (1:1, 0.2 M sodium cacodylate buffer, pH 7.3 with 2.5% glutaraldehyde) for 20 min. Then, 10 µL of the samples were dropped on a copper grid (300 mesh sized) covered with Formvarfor 2 min. 5% uranylacetate was used for contrasting. Images were captured on a Tecnaitransmission electron microscope at 120 kV (FEI) under conventional transmission mode with magnifications of 160,000 and 305,000x.

### Determination of viral antigenicity

Purified ZIKV^BR^ or CHIKV were treated with 300 µM CoPPIX and SnPPIX for 1 hour at 37 °C in the dark. In order to assess general virus reactivity, enzyme linked immunosorbent assay (ELISA) plates were coated overnight at 37 °C with 50 ul of untreated and treated virus in a final protein concentration of 2 µg/ml in PBS and blocked with PBS-T 1% BSA (Bovine Serum Albumin) for 2 hours at 37 °C. To determine specific E protein reactivity, ELISA plates were first coated with purified anti-E protein of Flavivirus antibody (Merck-Millipore, MAB102016) or anti-alphavirus E protein mouse monoclonal antibody (Merck-Millipore, MAB8754) with a dilution of 1:500 in carbonate-bicarbonate buffer (15 mM Na_2_CO_3_, 35 mMNaHCO_3_, pH 9.6) for 2 hours at 37 °C, blocked with PBS-T 1% BSA (Bovine Serum Albumin) for 2 hours and then incubated with the 4 × 10^3^ pfu/well of untreated and treated virus overnight at 37 °C. In both experiments, plate wells were incubated with anti-CHIKV or anti-ZIKV positive human sera serially diluted from 1:100 to 1:100000 for 2 hours at 37^o^ C for reactivity detection. A non-immune serum was also used as negative control and for determines background of reactivity. Serum samples were a kind gift from Dr. Orlando C. Ferreira Jr. (Instituto de Biologia, UFRJ). After, they were incubated with 0.16 µg/ml HRP-conjugated goat anti-human IgG antibody for 1 h at 37 °C. Washes with PBS-T (PBS with 0.05% Tween-20) were performed between all ELISA steps. Reactivity was developed with TMB substrate and read at 475 nm.

### Statistical analyses

All data are representative of at least three independent experiments performed in triplicate. Non-parametrical (Mann-Whitney) t-test was used to compare two groups at a time. To compare multiple groups one-way analysis of variance (ANOVA) was used with Bonferroni’spost test. Data were shown as means ± standard error (SEM) (with *p < 0.05, **p < 0.01, ***p < 0.001). Statistical analyses were performed with GraphPad Prism (version 6.0) software (GraphPad Software e Inc., La Jolla, CA).

### Data availability

All data generated or analyzed during this study are included in this published article (and its Supplementary Information files).

## Electronic supplementary material

Supplementary information
