# Supplementary material for: Co-protoporphyrin IX and Sn-protoporphyrin IX inactivate Zika, Chikungunya and other arboviruses by targeting the viral envelope
Source: Sci Rep. 2018 Jun 28;8:9805. doi: 10.1038/s41598-018-27855-7 (PMC6023862; doi:10.1038/s41598-018-27855-7)

Co-protoporphyrin IX and Sn-protoporphyrin IX inactivate Zika, Chikungunya and other arboviruses by targeting the viral envelope

Romulo L Neris[1]; Camila M Figueiredo[1]; Luiza M Higa[3]; Daniel F Araujo[1]; Carlos AM Carvalho[2]; Brunno RF Verçoza; Mariana OL Silva[1]; Fabiana A Carneiro[4], Amilcar Tanuri[3]; Andre MO Gomes[2]; Marcelo T Bozza[1],Andrea T Da Poian [2];Christine Cruz-Oliveira[2,*,+]; IranaiaAssunção-Miranda[1,*,+].

1. Instituto de Microbiologia Paulo de Goes, Centro de Ciências da Saúde, Universidade Federal do Rio de Janeiro (UFRJ).
2. Instituto de Bioquímica Médica Leopoldo de Meis, Centro de Ciências da Saúde, Universidade Federal do Rio de Janeiro (UFRJ).
3. Instituto de Biologia, Universidade Federal do Rio de Janeiro (UFRJ).
4. NUMPEX - Núcleo Multidisciplinar de Pesquisas, Polo Avançado de Xerém, Universidade Federal do Rio de Janeiro (UFRJ), Duque de Caxias, RJ, Brazil

5. Seção de Arbovirologia e Febres Hemorrágicas, Instituto Evandro Chagas (IEC), Ananindeua, PA, Brazil.
6. Departamento de Morfologia e Ciências Fisiológicas, Centro de Ciências Biológicas e da Saúde, Universidade do Estado do Pará (UEPA), Belém, PA, Brazil.

*Correspondence: Iranaia Assunção-Miranda, iranaiamiranda@micro.ufrj.br , Phone: 55 21 39388344 and Christine Cruz-Oliveira, ccruz@bioqmed.ufrj.br, Phone: 55 21 39386758

[+]These authors contributed equally to this work

Supplementary information

Supplementary figure 1: CoPPIX, SnPPIX and heme treated virus did not induce cell death. (A) $ZIKV_{BR}$, (B) $ZIKV_{766}$, (C) CHIKV, (D) MAYV, (E) SINV and (F) VSV were previously incubated with 300 μM CoPPIX, SnPPIX or heme without light-stimuli (black bars) or 10 μM of SnPPIX under light-stimuli (white bar). After, cells were infected with porphyrin-treated or non-treated virus using a MOI of 0.1. Cell viability was measured by MTT assay. Data are represented as means ± SEM. of at least three independent experiments. Results are statistically significant with $*p \leq 0.05$ or $**p \leq 0.01$.

Supplementary figure 2: Absence of envelope protein synthesis after infection with CoPPIX and SnPPIX treated virus. Cells were infected with untreated or treated-$ZIKV^{BR}$, CHIKV, MAYV and SINV with 300 μM CoPPIX or 10 μM SnPPIX under light-stimuli (LS). Viral E protein detection was assessed 24 h post infection by fluorescence microscopy. The corresponding bright field images for each condition are also shown. Images were taken with magnifications of 10x.

Supplementary figure 3: **Full-length SDS-PAGE of purified virus particles after treatment with porphyrins**. Protein profile of purified(A) $ZIKV^{BR}$,(B) CHIKV (C) MAYV and (D) VSV by 10% SDS-PAGE. Viral particles were treated with 300 μM CoPPIX, SnPPIX or heme without or with light stimuli (LS). Treatment conditions are identified and viral proteins are indicated for each virus.

Supplementary Figure 1

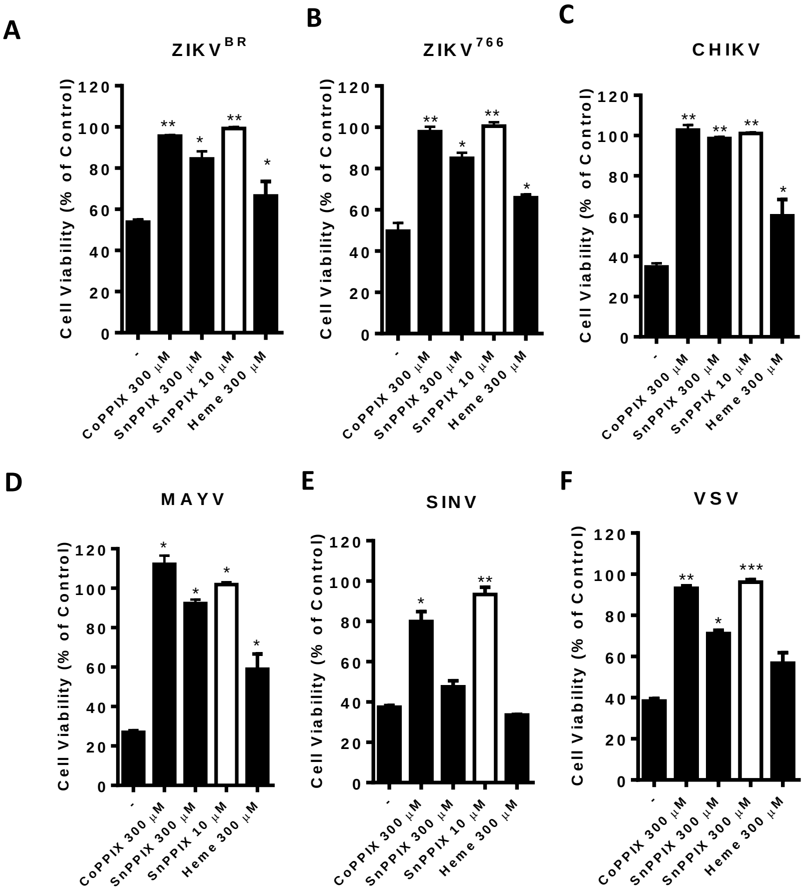

Supplementary figure 2

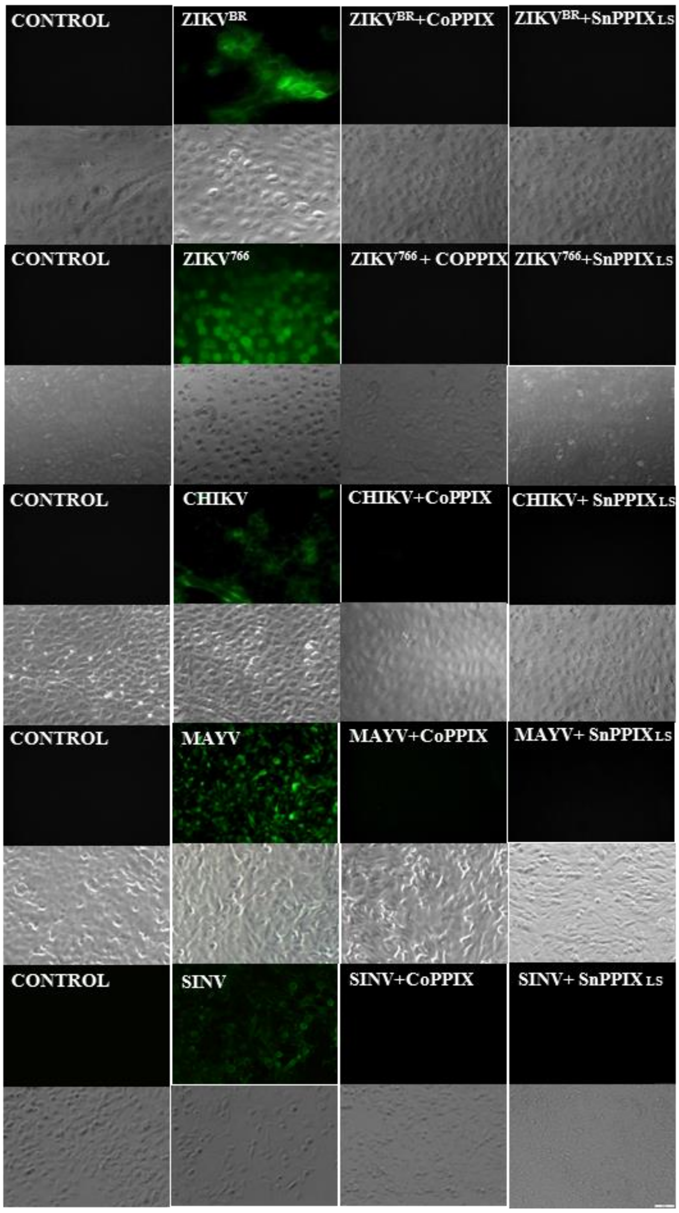

Supplementary Figure 3

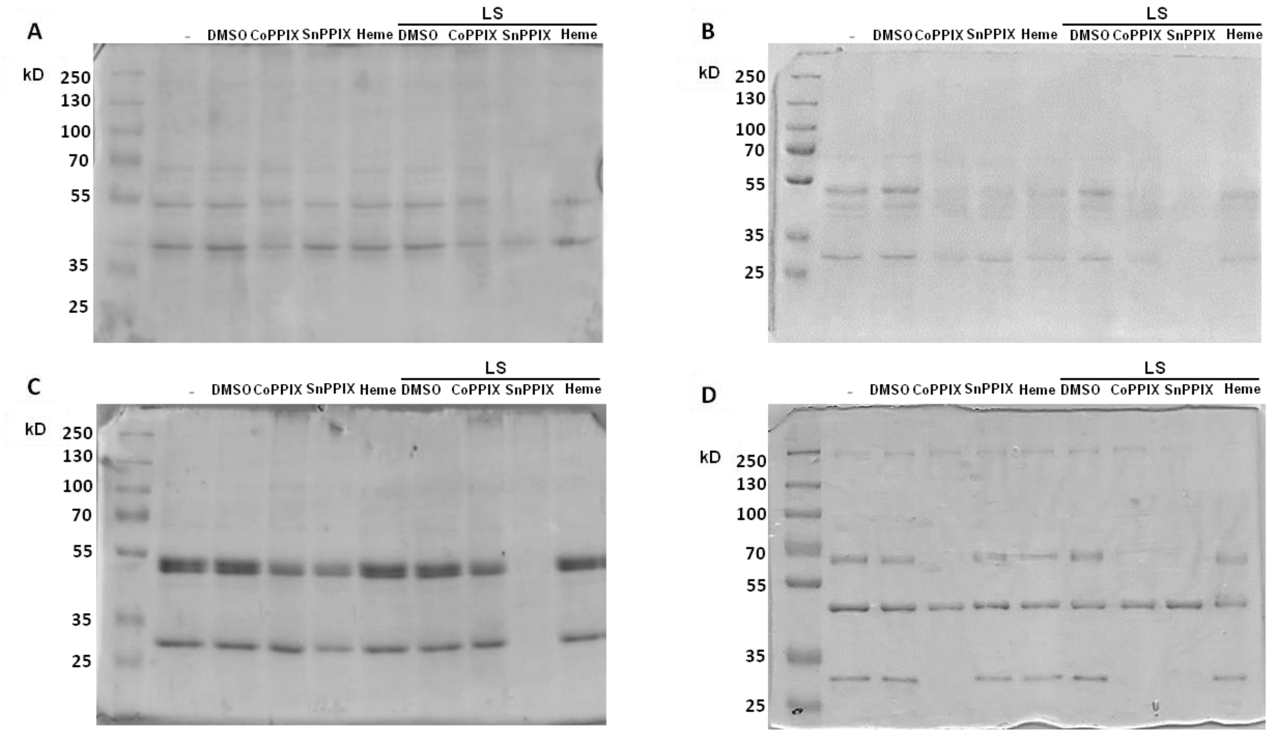

Supplement: Supplementary file 1 — Supplementary information [file 41598_2018_27855_MOESM1_ESM.pdf]
